# Supplementary material for: Mitochondrial Fusion Via OPA1 and MFN1 Supports Liver Tumor Cell Metabolism and Growth
Source: Cells. 2020 Jan 4;9(1):121. doi: 10.3390/cells9010121 (PMC7017104; doi:10.3390/cells9010121)
Supplement: Supplementary file 1 [file cells-09-00121-s001.pdf]

Supplementary data for

Mitochondrial fusion via OPA1 and MFN1 supports liver tumor cell metabolism and growth

Meng Li, Ling Wang, Yijin Wang, Shaoshi Zhang, Guoying Zhou, Ruby Lieshout, Buyun Ma, Jiaye Liu, Changbo Qu, Monique M. A. Verstegen, Dave Sprengers, Jaap Kwekkeboom, Luc J. W. van der Laan, Wanlu Cao, Maikel P. Peppelenbosch, Qiuwei Pan

Table of contents

Supplementary Figure 1

Supplementary Figure 2

Supplementary Figure 3

Supplementary Figure 4

Supplementary Figure 5

Supplementary Figure 6

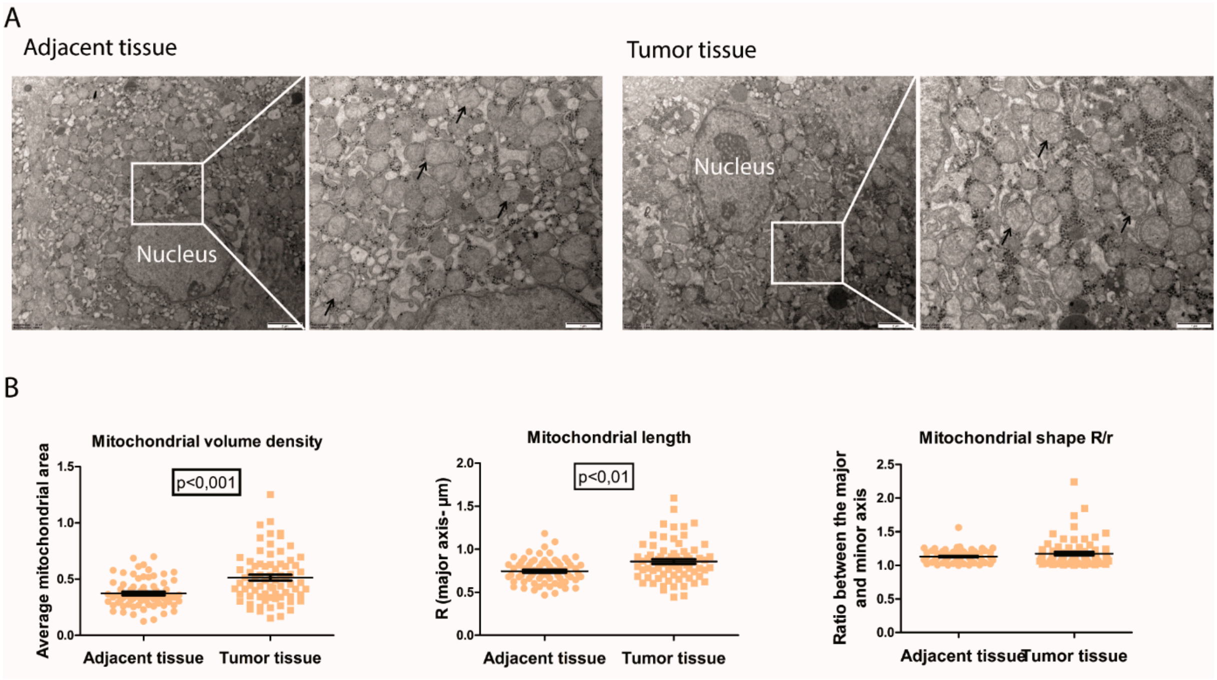

Supplementary Fig 1 Mitochondrial morphology in a HBV-related HCC patient. **A** Representative electron microscopy images of cells from adjacent tissue and tumor tissue of the HCC patient (scale bar = 2 μm). The white boxed regions were further magnified in the expanded images (right side) (scale bar = 1 μm). Mitochondria (arrows) were observed. **B** Images of paired tissue were analysed by ImageJ software (n = 5 images/sample). Mitochondrial volume (mitochondrial area) per cell and mitochondrial length (mitochondrial major axis) were valued. Mitochondrial shape was calculated by the ratio of major axis and minor axis. Histograms are mean ± SEM, with *p* values by Mann Whitney test.

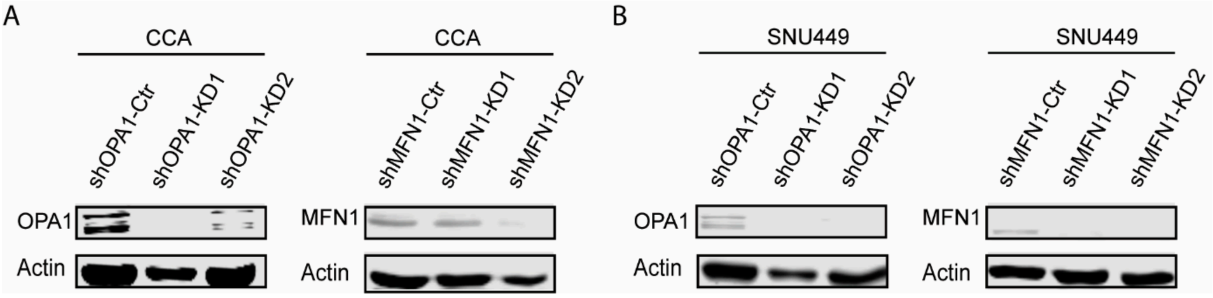

Supplementary Fig 2 Western blot analysis of mitochondrial fusion genes in liver cancer cells SNU449 and CCA organoids. **A** OPA1/MFN1 protein level of CCA organoids with OPA1/MFN1 downregulation. **B** OPA1/MFN1 protein level of SNU449 cells with OPA1/MFN1 downregulation.

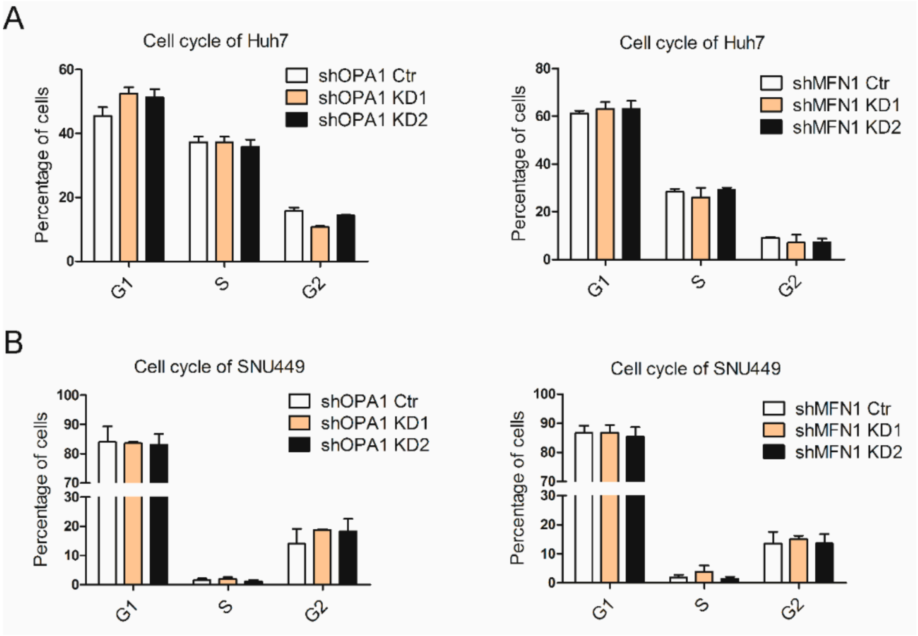

Supplementary Fig 3 Dysfunction of mitochondrial fusion inhibited tumor cell growth independent of cell cycle arrest. **A** The distribution of Huh7 cells with OPA1/MFN1 downregulation did not show significantly difference with control group (n = 3). **B** The distribution of SNU449 cells with OPA1/MFN1 downregulation did not show significantly difference with control group (n = 3). Histograms are mean ± SEM.

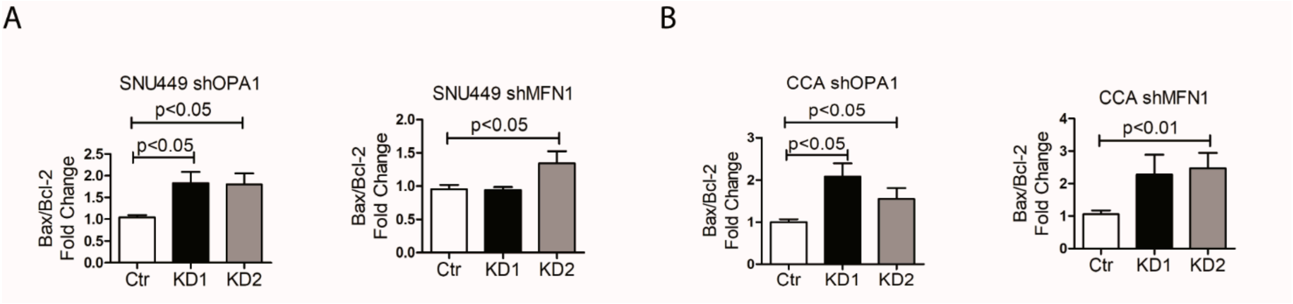

Supplementary Fig 4 Dysfunction of mitochondrial fusion is correlated with increased ratio of Bax/Bcl-2 gene expression in liver cancer cells. **A** Bax/Bcl-2 fold change of SNU449 cells with OPA1/MFN1 downregulation increased compared with control group (n = 6). **B** Bax/Bcl-2 fold change of CCA organoids with OPA1/MFN1 downregulation increased compared with control group (n = 6). Histograms are mean ± SEM, with *p* values by Mann Whitney test.

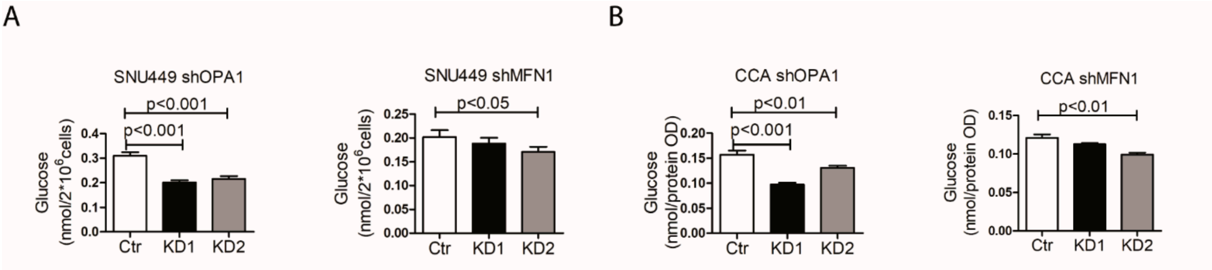

Supplementary Fig 5 Dysfunction of mitochondrial fusion down-regulated glucose level in liver cancer cells. **A** The glucose product level of SNU449 cells with OPA1/MFN1 downregulation showed decreased glucose level compared with control group (n = 6). **B** The glucose product level of CCA organoids with OPA1/MFN1 downregulation showed decreased glucose level compared with control group (n = 6). Histograms are mean ± SEM, with *p* values by Mann Whitney test.

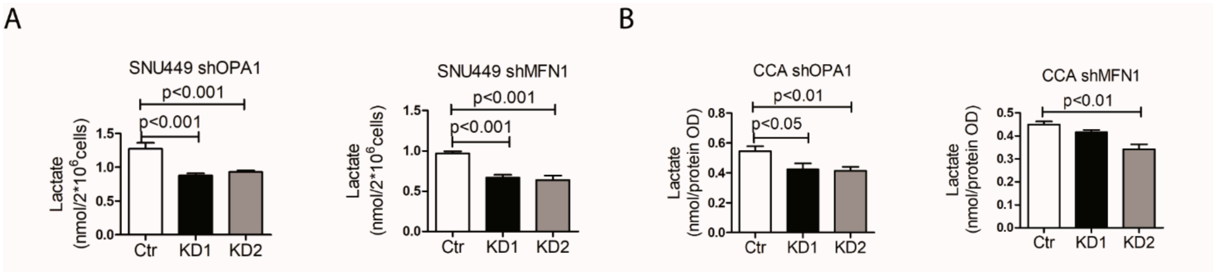

Supplementary Fig 6 Dysfunction of mitochondrial fusion down-regulated lactate level in liver cancer cells. **A** The lactate product level of SNU449 cells with OPA1/MFN1 downregulation showed decreased lactate level compared with control group (n = 6). **b** The lactate product level of CCA organoids with OPA1/MFN1 downregulation showed decreased lactate level compared with control group (n = 6). Histograms are mean ± SEM, with *p* values by Mann Whitney test.
